# Agents for sequential learning using multiple-fidelity data

**DOI:** 10.1038/s41598-022-08413-8

**Published:** 2022-03-18

**Authors:** Aini Palizhati, Steven B. Torrisi, Muratahan Aykol, Santosh K. Suram, Jens S. Hummelshøj, Joseph H. Montoya

**Affiliations:** 1 Energy and Materials Division, Toyota Research Institute, Los Altos, USA; 2 Department of Chemical Engineering, Carnegie Mellon University, Pittsburgh, USA

**Keywords:** Chemistry, Energy science and technology, Engineering, Materials science, Mathematics and computing, Optics and photonics

## Abstract

Sequential learning for materials discovery is a paradigm where a computational agent solicits new data to simultaneously update a model in service of exploration (finding the largest number of materials that meet some criteria) or exploitation (finding materials with an ideal figure of merit). In real-world discovery campaigns, new data acquisition may be costly and an optimal strategy may involve using and acquiring data with different levels of fidelity, such as first-principles calculation to supplement an experiment. In this work, we introduce agents which can operate on multiple data fidelities, and benchmark their performance on an emulated discovery campaign to find materials with desired band gap values. The fidelities of data come from the results of DFT calculations as low fidelity and experimental results as high fidelity. We demonstrate performance gains of agents which incorporate multi-fidelity data in two contexts: either using a large body of low fidelity data as a prior knowledge base or acquiring low fidelity data in-tandem with experimental data. This advance provides a tool that enables materials scientists to test various acquisition and model hyperparameters to maximize the discovery rate of their own multi-fidelity sequential learning campaigns for materials discovery. This may also serve as a reference point for those who are interested in practical strategies that can be used when multiple data sources are available for active or sequential learning campaigns.

## Introduction

A central concern of the materials discovery and optimization process is a simple, practical question: given limited researcher time and resources, what is the next experiment that should be performed? The urgent need for new energy technologies to mitigate fossil fuel use makes this question especially relevant. Widespread adoption of novel fuel cell catalysts, batteries, thermoelectrics, and other energy technologies requires optimization on many different fronts: materials discovery campaigns may target compounds with improved cost, safety, stability, efficacy, or some combination of these and other goals. The use of artificial intelligence tools to accelerate the discovery and optimization process, hand-in-hand with developments in high-throughput experimentation and analysis, may help us to meet timely goals for decarbonization of the global energy economy.

This work is a step towards bridging three relatively recent advances in the materials science research community, which are still realizing their individual and combined potential: (1) the advent of large-scale and freely available databases of computational simulations^1–4^, particularly from density functional theory (DFT)^5,6^, (2) the mainstream accessibility of machine learning tools^7^, and (3) development of high-throughput experimentation hardware and software^8–11^. DFT has shown its applicability in complementing and even guiding the experimental discovery of materials^12–14^. Machine learning exploits the widespread availability of DFT results to allow accurate and interpretable surrogate models to estimate a desired materials property before either experiment or simulation^12,15–23^. By increasing the efficiency of theoretical property prediction, the combination of large-scale DFT and machine learning makes it easier for researchers to obtain theoretical predictions for a wider variety of materials, which can then guide the high-throughput experimental process. The paradigm of sequential (or active) learning (henceforth SL), in which a model solicits new training data and updates its performance in response to this data, is useful both to computational^24–28^ and experimental high throughput studies for both optimization and analysis^29^. Some examples of sequential learning include: systems that learn how to perform only the most valuable or relevant DFT simulations using previous iterations^25,30,31^, improve force fields more rapidly for molecular dynamics simulations^24,32^, and synthesize carbon nanotubes at new conditions that promote higher yields and higher qualities of product^33^. The sequential learning paradigm thus can provide a conceptual link between materials optimization and discovery workflows across computational and experimental methodologies.

This work also considers a complication in the reality of the scientific discovery process, where there are many sources of data with different costs to obtain them. We use “multi-fidelity” to describe these diverse data sources, assuming a tradeoff between data accuracy and cost to obtain. High-fidelity data is considered expensive to obtain but more accurate, whereas low fidelity data is considered cheaper to obtain but less accurate. A promising approach to utilize data with various fidelity is through multi-fidelity models. These models provide a clear conceptual advantage: they can combine large quantities of cheaply acquired, less accurate data with data acquired via more expensive but accurate methods. Multi-fidelity models mitigate resource limitations of building models from high fidelity data and can be significantly more accurate in their predictions than similar models trained on single-fidelity datasets^34–38^. For example, multi-fidelity models can combine theoretical calculations (such as DFT data) with experimental observations, or compare DFT calculations with a computationally efficient functional like PBE with a more costly but accurate functional like HSE^39,40^ or SCAN^41,42^. Currently, multi-fidelity modeling methods are typically used and investigated independently from sequential learning in the scientific literature, i.e. the relationship between the acquisition strategy and the modeling strategy is chosen on an ad-hoc basis. This work explores and attempts to show how scientists can use sequential learning and multi-fidelity methods together.

In this work, we introduce a multi-fidelity sequential learning framework for materials discovery based on agents which minimize the number of high fidelity acquisitions while simultaneously optimizing for a figure of merit. These agents are open-source and new additions to the existing software framework CAMD^31^. We demonstrate the agents’ capability using electronic band gap data of inorganic compounds at two fidelities: experimental measurements as high fidelity and GGA-level DFT simulations as low fidelity. We choose the band gap as it is a fundamental electronic property relevant for a wide range of technological applications^14,43,44^. Hence, materials with targeted bandgaps have been the subject of several combinatorial high-throughput efforts including searches for photocatalytic materials with the capability of absorbing sunlight^45–47^. Thus, there is a large body of experimental and theoretical bandgap data covering a wide range of chemical systems^35,36,48^. Using our framework and this previously-existing data, we performed several iterative sequential learning campaigns to ‘simulate’ the discovery of inorganic materials with a target electronic band gap window. The scope of this study is thus to benchmark the process of integrating multiple fidelities in a discovery campaign. This study serves as “wargames” for multi-fidelity sequential learning campaigns so that in applying these tools to a real-world campaign (with its associated costs on researcher time and resources), the agents can be well-chosen for the task with a greater degree of confidence and user knowledge.

We benchmark discovery campaigns in two settings: one where all the low-fidelity or “cheap” data is available from the start, and one where low-fidelity data is acquired in parallel to high-fidelity data. In other words, in the first setting, DFT data is considered prior knowledge, and in the second setting, DFT and experimental data are acquired together in sequential iterations. These sequential learning procedures benchmark how different ML models and acquisition strategies influence the overall rate of discovery of materials per experiment, which was scored using several previously used figures of merit^49^. Our results show that consideration of lower fidelity DFT data in conjunction with experimental data increases the rate of discovery of materials suitable for solar photoabsorption, which suggests that this may be a useful strategy for high-throughput campaigns involving variable data fidelity to use to accelerate their work. We further demonstrate that the type of machine learning model used in the sequential learning procedure controls the extent of an increase in the number of discoveries in the multi-fidelity setting compared to the single-fidelity baseline.

## Methods

Below, we will detail how we compiled and encoded the band gap database in “Dataset collection and representation”, how we designed a sequential learning procedure that can use and request data in “Multi-fidelity sequential learning procedure”, how we developed agents which can account for this additional complexity in “Agent design for materials discovery”, how we evaluated the agents in “Performance metrics for sequential learning”, and what our objective for our benchmarking campaigns is in “Sequential learning objective”.

### Dataset collection and representation

**Figure 1:**
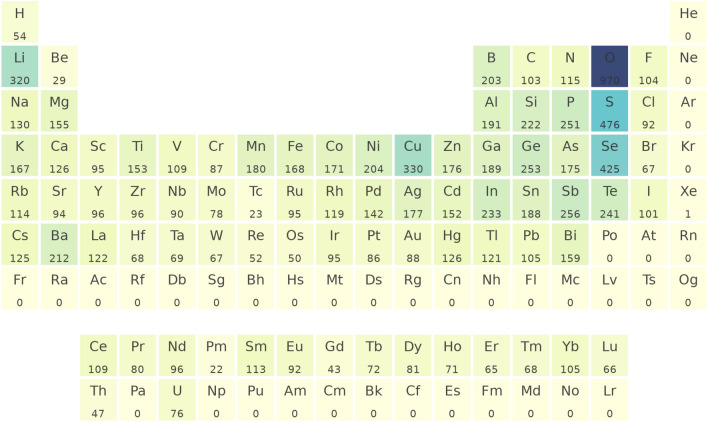
The periodic table outlining frequencies of the elements occurring in our band gap dataset. The color represents the frequency of occurrence. This frequency is also labeled under each element symbol.

The band gap dataset was collected from two sources: (1) experimentally reported band gaps of inorganic semiconductors aggregated by Zhuo et al.^34^ and disseminated via the Matminer^50^ package, and (2) GGA-level DFT-computed band gaps generated and disseminated via the Materials Project database^1,51^. We first pulled the experimentally reported compositions and their corresponding band gaps. For each experimental composition, we attempted to obtain the band gap corresponding to the most phase-stable (i.e. lowest computed energy per atom) crystal structure from the Materials Project. Here, the DFT-computed band gaps using the Perdew–Burke–Ernzerhof (PBE) functional^52^ were considered low fidelity data, as GGA has well-known systematic errors that underestimate experimentally measured band gaps by $\sim$ 0.9 eV^53^. Overall, 3960 unique compositions had both experimental and theory data. Out of the 3960 compositions, 375 contained multiple experimental band gap measurements. For each composition with multiple experimental measurements, the respective minimum band gap value was used. Figure 1 describes the dataset by outlining the element occurrence, which shows abundant oxides, sulfides, and selenides, as well as copper and lithium-containing compositions.

We processed the collected data by using a fixed-length vector to encode both the compositions of each material and the level of fidelity. More specifically, stoichiometric compositions were featurized with the matminer ElementProperty featurizer^50^. This featurizer offers flexibility to compare experimental and theoretical data when experimental structure information is not available. The levels of fidelity were represented with one-hot encoding, where a binary variable was added for each fidelity level (i.e. for experimental data, a “1” was placed under the “experiment” feature and a “0” under the “theory” feature and vice versa). To improve numerics, the final overall features were scaled such that their distribution had a mean of 0 and a standard deviation of 1.

### Multi-fidelity sequential learning procedure

The multi-fidelity sequential learning framework is built on the recently introduced system for Computational Autonomy for Materials Discovery (CAMD)^31^. CAMD is a framework that abstracts decision-making in sequential learning studies into “agents”. Agents perform tasks like training and applying machine learning models or choosing which experiment should be done next based on user-specified criteria. CAMD is open-source and users can add new agents according to their needs (such an agent is one of the contributions of this manuscript). The CAMD framework enables convenient design and testing of acquisition strategies from candidate data points in SL-based optimization.

**Figure 2:**
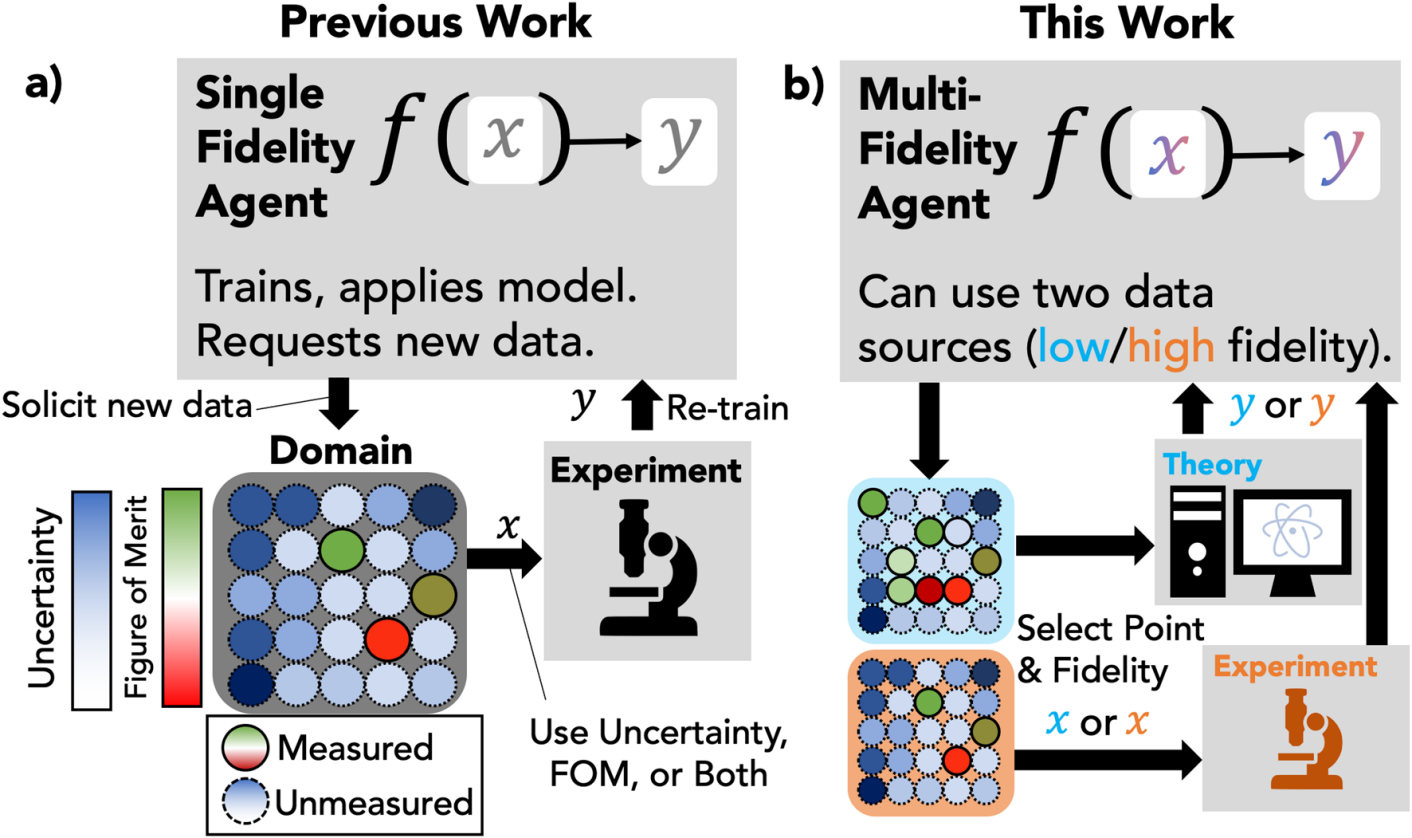
A schematic outlining the distinguishing features of our new multi-fidelity sequential learning framework. (**a**) Previous work established in Ref^31^. The major components of the framework include an agent, which maintains a model which is iteratively updated in response to new data selected and solicited by the agent. The agent passes the instruction to an “experiment” (a generic term for some kind of data generation, and could alternately be a theoretical calculation). (**b**) Our work is distinguished by making available two different sources of data with differing degrees of fidelity (here, we use experimental and theoretical data). The agent makes predictions using both sources of data and makes the decision to select new data from one pool or the other.

Figure 2 outlines the CAMD framework and highlights the newly constructed multi-fidelity acquisition feature. In a given series of iterations, termed a *campaign*, the (multi-fidelity) seed data and candidate data (search space) go into an *agent*. A preprocessing step in the agent featurizes each data point in the *seed data* and *candidate data* using the point’s composition and fidelity as described in the previous section. The featurized seed data is used to train a machine learning model, which makes predictions on the candidate data for the target property. Using the predictions, the agent then selects candidates at different fidelities. In the CAMD framework, candidates selected by the agent are sent to an *experiment* API, which collects the experimental data corresponding to the candidate and augments the dataset, allowing candidate data to be moved into the seed data for new active learning iterations. For the sake of active learning simulation, the CAMD experiment is an “after-the-fact” (ATF) API that emulates DFT simulation and experimental measurement, respectively, and returns the results from the known dataset which the agent and CAMD campaign are not aware of prior to the acquisition. This after-the-fact protocol reflects the scope of our study: to benchmark multi-fidelity agent performance in how efficiently they explore a known dataset, demonstrating the gains of multi-fidelity agents with various exploration strategies. The ATF experiment API can be exchanged for one that collects data from and monitors a real experiment, performing new experiments or DFT simulations with the agent that has been designed using ATF simulations^31^. Another CAMD object, the *analyzer*, monitors the campaign results and provides an analysis of the experiments in the context of the previously collected data (i.e. the seed data) and the progress of the campaign. In our case, the analyzer monitors and reports the cumulative number of materials suitable for solar photoabsorption. Upon the completion of the agent selection, experimental acquisition, and analysis phases of a campaign iteration, newly obtained experimental results are appended to the seed data and removed from the candidate data, and campaign begins in a new iteration with agent selection.

### Agent design for materials discovery

Designing the agent for a multi-fidelity sequential learning procedure required two steps: (1) selecting appropriate machine learning models and (2) generalizing a CAMD-compatible^31^ data acquisition decision-making process to allow for multiple levels of data fidelity. For model selection, we implemented and compared several well-known regression methods, including support vector regression (SVR), k-nearest neighbors (KNN), random forest regression (RFR), and Gaussian process regression (GPR). For each model, we optimized hyperparameters and did comparative performance analysis (detailed results can be found in Supplementary document S1). Based on the results, support vector regression, random forest regression, and Gaussian process regression had qualitatively similar performances and were used for framework construction and demonstration. Our implementation is sufficiently general to allow users to choose any scikit-learn-compatible ML model and their choice of hyperparameters.

A primary design concern in developing a multi-fidelity agent is mathematically framing the problem of when to draw from low-cost, low-fidelity data vs. high-cost, high-fidelity data. To this end, we designed two agents, an epsilon-greedy multi-fidelity agent (henceforth $\epsilon$-greedy-MF) and a Gaussian process lower confidence bound^54^ derived multi-fidelity agent (GPR$_{LCB}$-MF). The latter exploits the fact that Gaussian Process regression allows for a principled uncertainty estimate “out-of-the-box”, whereas the former works for regression algorithms lacking this feature.

The salient features of the $\epsilon$-greedy agent are that it takes as input a budget of high-fidelity datapoints *n* which controls the balance between low-fidelity and high-fidelity data. The agent will only call for high-fidelity measurements in domains that have been previously covered by low-fidelity data (see details in Algorithm S1). The $\epsilon$-greedy-MF agent works using any supervised machine learning regressor from scikit-learn^7^ as input. Meanwhile, the GPR$_{LCB}$-MF agent operates under a total acquisition budget and calls for low- or high-fidelity data in a more sophisticated way. It acquires candidates factoring in Gaussian process regression predicted uncertainties in the LCB setting and *hallucination* of information gain from low fidelity acquisitions analogous to work of Desautels et al. in batch mode LCB^55^ (see full details in Algorithm S2). *Hallucination* works as such: for a high fidelity candidate, the GPR$_{LCB}$-MF agent adds the lower fidelity predicted posterior mean into the seed data. As a consequence, the higher fidelity candidate prediction gets updated. Essentially, hallucination refers to the ability of the agent to predict ahead of time how low-fidelity data will impact the uncertainty estimate of the model. Hallucination allows the agent to use low-fidelity candidates to explore potentially promising parts of the domain, while using high-fidelity candidates to exploit promising regions of parameter space, offloading exploratory (higher risk) acquisitions first to lower-fidelity computations. In our formulation, three hyperparameters that must be empirically optimized govern the tradeoff between data fidelities: $\alpha$, $\beta$, and $\gamma$. $\alpha$ is the uncertainty multiplier in GPR$_{LCB}$ as shown below:

1 $$\begin{aligned} \hat{y_i}_{,LCB} = \hat{y_i} - \alpha * \sigma _i \end{aligned}$$

where $\hat{y_i}$ is the posterior mean and $\sigma _i$ is the uncertainty given a candidate i. $\alpha$ here sets the weight of uncertainty in the LCB setting. Next, $\beta$ is a threshold for uncertainty. For a given observation, if its $\sigma _i$ is less than $\beta$, the observation is considered to have low uncertainty. A small $\beta$ makes the agent “risk-averse” around high-fidelity measurements in unexplored regions of space. In the small $\beta$ regime, unless the uncertainty on a given prediction is very low, it will acquire lower fidelity data first. Inversely, if $\beta$ is large, the agent is tolerant to high uncertainty for experiments and will more readily add experimental data. In practical applications, $\beta$ could be set with respect to the cost of acquiring high-fidelity data. Lastly, $\gamma$ is a threshold for the influence of hallucination (denote $\Delta r$) as shown below:

2 $$\begin{aligned} \Delta r = r^*_i - r_i \end{aligned}$$

**Figure 3:**
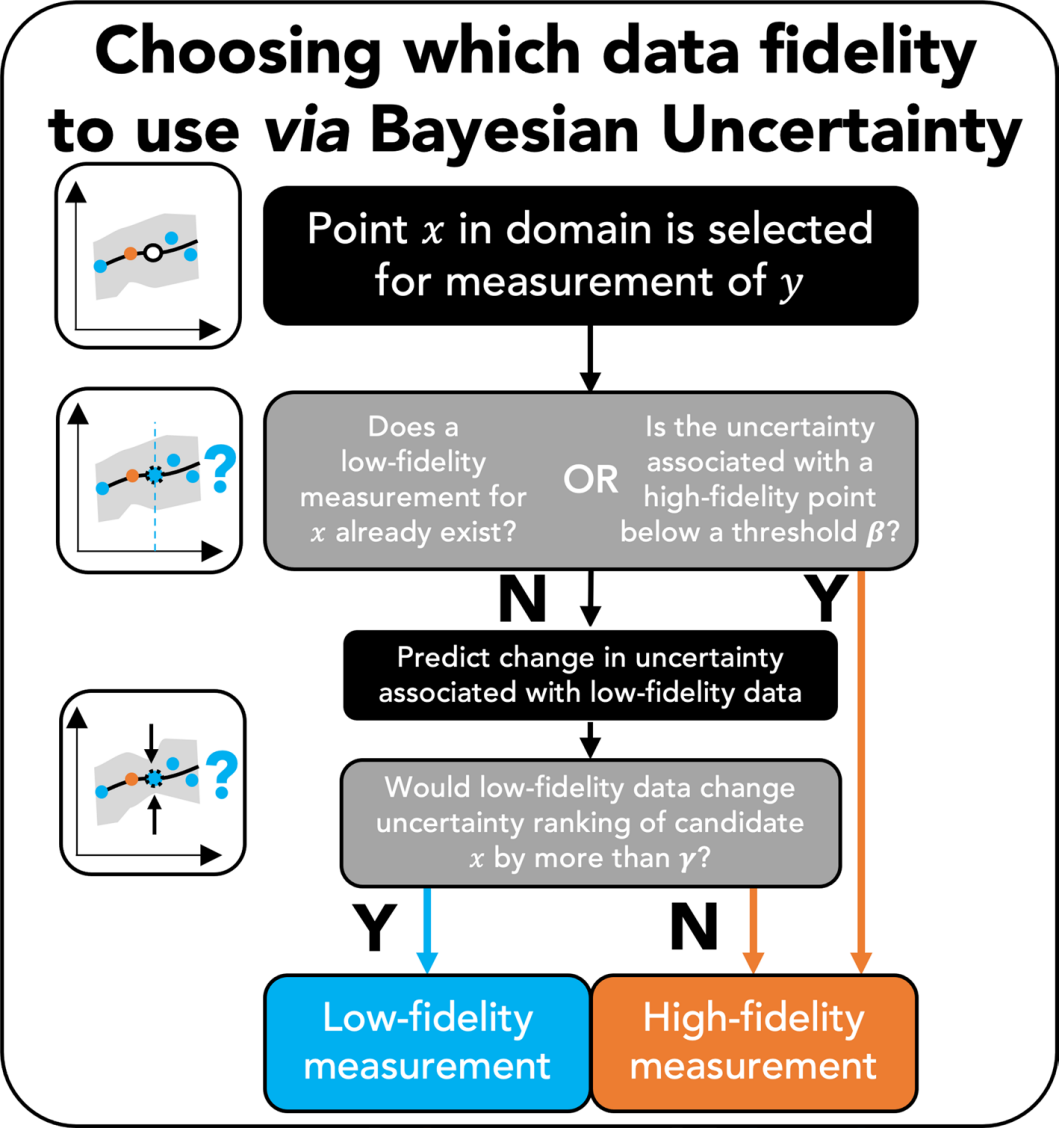
Overview of how uncertainty is used to balance the use of low and high fidelity data in the implementation of Algorithm 2 of Section S2. When a data point *x* is called for as an experimental candidate, the above flowchart describes the decision-making process for which data source to use. After a data point *x* is selected for measurement, two conditions are checked: (1) if the corresponding (e.g. with the same formula) low-fidelity measurement has already been made or (2) if the uncertainty associated with the high fidelity measurement is low enough (below a threshold $\beta$). If either is true, the high-fidelity measurement is taken. If neither are true, then the agent must consider the trade-off between low-fidelity and high-fidelity data. The agent thus considers how a low fidelity data point would affect the current ordering of the predicted figure of merit associated with all candidates. If it would alter the ranking by more than $\gamma$, the low-fidelity measurement is taken. If not, the high-fidelity measurement is taken. Note that $\beta$ and $\gamma$ are user-defined hyperparameters explained in detail in Section S2.

Here, $r_i$ is the ranking of an observation $\hat{y_i}_{,LCB}$ in the candidate space based on its distance to the target value. $r^*_i$ is the new ranking of the observation after hallucination.The agents acquire a high fidelity candidate if $\Delta r \le \gamma$. If $\gamma$ is 0, then the prospect of the lower fidelity data has to increase the chances of the experiment being successful. Otherwise, lower fidelity data will be acquired first. Because in this case, r$^*_i$ has to be a smaller value than r$_i$ (i.e. a better ranking). If $\gamma$ is very large, then the agent does not care about how much low fidelity data affects the potential experiment. Because in this case, r$^*_i$ can be any value, including a value that is higher than r$_i$ (i.e. a worse ranking). The overall influence of these hyperparameters is summed up in a broad overview way in Fig. 3. We simulated various scenarios of these three hyperparameters to optimize the agents. The details and results are in Supplementary document section S3. The Gaussian processes were implemented using the GPy^56^ package. Details of agents are also made explicit in the code available via the open-source CAMD repository at https://github.com/TRI-AMDD/CAMD.

### Performance metrics for sequential learning

To quantitatively compare the efficacy (the ability to call for data points that optimize a figure of merit) and efficiency (the number of experiments required to do so) between active learning campaigns, we used previously established active learning metrics (ALM) described by Rohr et al.^49^. These metrics are ALM, acceleration factor (AF), and enhancement factor (EF), defined as follows and explicated further below:

3 $$\begin{aligned} ALM(x, N_{exp}) = \frac{\text {Target Materials Discovered after }N_{exp}}{\text {Total Target Materials}} \end{aligned}$$

4 $$\begin{aligned} AF(x,y,ALM) = N_{exp}(x|ALM) - N_{exp}(y|ALM) \end{aligned}$$

5 $$\begin{aligned} EF(x,y,N_{exp}) = ALM(x,N_{exp}) - ALM(y,N_{exp}) \end{aligned}$$

where *x* and *y* are agents, $N_{exp}$ is the number of experiments performed (i.e. in our case, the high fidelity data acquired). The function $N_{exp}$ conditioned on ALM (i.e. N$_{exp}$(x|ALM) and N$_{exp}$(y|ALM)) refers to the number of experiments in the sequential learning campaigns that attained an ALM. Because our emulated discovery campaigns are trying to find materials with a visible-spectrum band-gap, our discovery process can be scored in a binary way: any new data point’s band gap is either inside or outside of the target range. Thus, we can compute the fraction of ideal materials which were correctly identified at an iteration given a sequential learning run and so ALM lies within [0, 1]. This metric is defined for a single sequential learning campaign and is most useful for after-the-fact workflows, as the denominator requires some knowledge of the total number of materials which lie within the target range (For a ‘real-world’ case where the materials are not known ahead of time, the final number of target materials discovered by the campaign can be used in scoring sequential learning, as ALM is a ‘time-dependent’ property that can change at each iteration step. Also, note that this study focuses on materials that are scored in a binary way as having the band gap property within a target range. For cases where a quantity is optimized around some target, this could be defined using the distance of the best-known material thus far to the current best-known global maximum/minimum target property). Next, acceleration factor and enhancement factor are metrics that compare *two* sequential learning runs to one another using the ALM. The acceleration factor is the reduction of required budget (e.g. in time, iterations, or some other consumed resource) between an agent and a benchmark case (e.g. random selection, an alternate model, single-fidelity, or manual human selection) to reach a particular fraction of ideal candidates (AF = N$_{budget, benchmark}$ - N$_{budget, agent}$). In other words, given ALM *vs.* $N_{exp}$, the acceleration factor is the “horizontal-line” distance between two models at an ALM at different “times”. A positive value of acceleration factor between a multi-fidelity campaign and a single-fidelity campaign means the former outperformed the latter because it reduced the required budget needed to achieve a certain amount of discovery. Similarly, the enhancement factor is the “vertical-line” distance between two campaigns’ ALM score at a given “time”, which shows the performance enhancement at the same consumed experiment budget. More specifically, at the same number of iterations, amount of elapsed time, or some other metric of expended resources, enhancement factor quantifies the improvement of materials discovery by a given sequential learning method versus a benchmark method (EF = $\frac{N_{discovery, agent}}{N_{discovery, benchmark}}$). In the case of comparing a multi-fidelity campaign to a single-fidelity campaign, when the enhancement factor is greater than one, it indicates that the multi-fidelity campaign outperforms its corresponding single-fidelity campaign at a given budget.

### Sequential learning objective

For multi-fidelity sequential learning campaign simulations and subsequent performance evaluations of the agents, we attempted to model a discovery campaign for photoabsorbers by targeting materials with experimentally measured band gap $\subseteq$ [1.6, 2.0] eV^57^, i.e. those with reasonable solar photoabsorption, were considered ideal and set as the targets. 207 of our 3960 candidate experimental materials are considered ideal based on the target band gap window defined above. In other words, only about one in twenty or 5% of the candidate materials lie within the target window of the discovery campaign.

## Results

**Figure 4:**
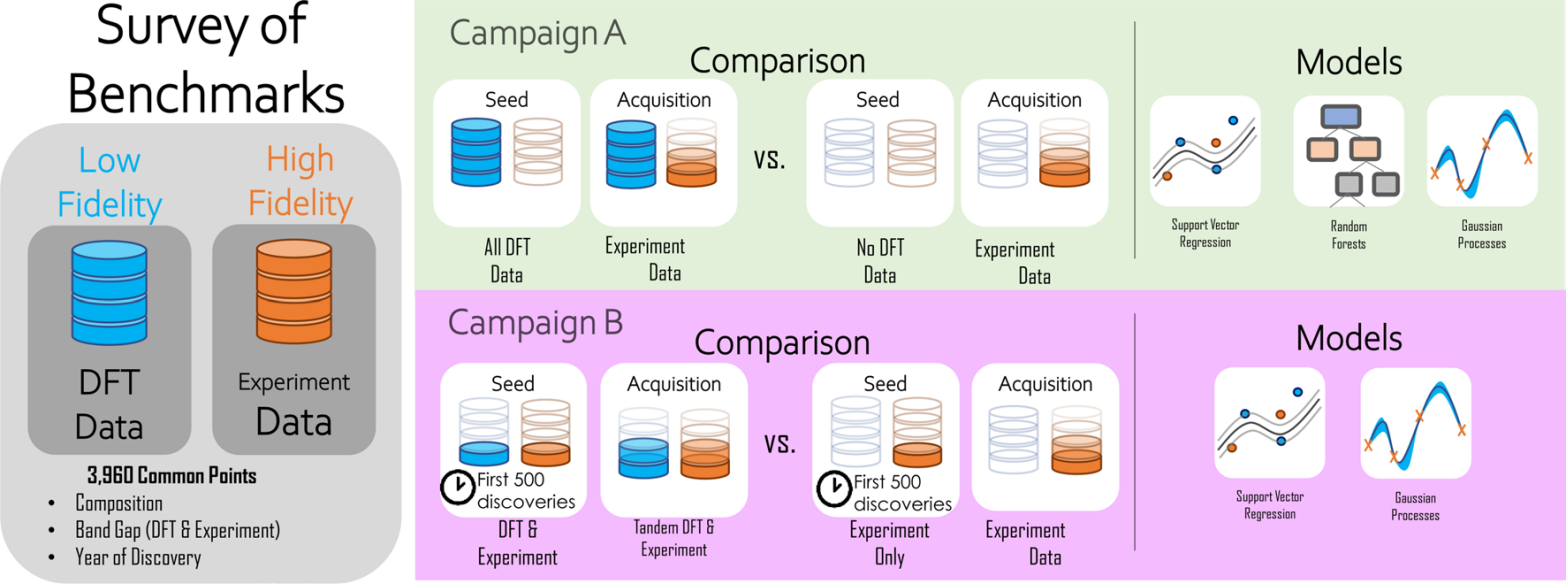
Campaigns performed for single-fidelity acquisition and multi-fidelity acquisition. In campaign A, we compare the results of including all or no DFT data in the seed data set and only acquire experimental data. This campaign effectively compares an “a priori” agents to a single-fidelity agents. In campaign B, the multi-fidelity agents are seeded with first 500 experimentally discovered compositions (based on ICSD^58^ timeline of their first publication^59^) and their corresponding DFT data. Both DFT and experimental data are acquired here. The single-fidelity agents are exclusively seeded with and acquire experimental data.

Figure 4 highlights the campaigns that we performed to benchmark the sequential learning models. In “Boundary cases: all or no DFT data available” (corresponds to campaign A), we demonstrate the performance gains which come from an agent with full a priori knowledge of DFT calculations soliciting experimental data versus an agent which never uses DFT data exploring the same space of experiments. Next, in “In-tandem acquisitions: both DFT and experiment data are acquired” (corresponds to campaign B), we compare the performance of agents seeded with first 500 experimentally discovered compositions in a multi-fidelity versus single-fidelity context, where either both DFT and experimental data are solicited in-tandem (with some DFT data supplied a priori) or exclusively experimental data seeded and solicited. In both cases, we find that the performance of multi-fidelity agents are improved by the inclusion of low-fidelity DFT data.

### Boundary cases: all or no DFT data available

We first tested the acquisition performance of multi-fidelity agents in the limiting case where the full suite of DFT calculations was considered as a priori knowledge. The objective here was to determine how an automated experimental sequential learning procedure would be enhanced by a priori knowledge of a large theoretical dataset. This type of acquisition is for a use case where low-fidelity experimental data is much cheaper to acquire than high-fidelity data that full domain coverage is available at the outset of a high-fidelity experimental campaign. Because no new low-fidelity data is solicited, gains in campaign performance are entirely due to the transfer of knowledge from the large, low-fidelity dataset in making predictions and subsequent acquisitions under the high-fidelity, expensive setting.

We performed after-the-fact discovery runs with three agents: $\epsilon$-greedy agents that used support vector regression and random forest regression, and a GPR$_{LCB}$ agent. As mentioned previously, $\epsilon$-greedy agents works for regression models lack principled uncertainty estimate, and GPR agent acquire candidates based on both the predicted posterior mean and uncertainty from Gaussian process regression. For each agent, we considered two cases: (1) no low-fidelity seed data at any point in the campaign and (2) all available DFT data as seed data at the outset. Note that both (1) and (2) are thus only acquiring high-fidelity data, and this set of six campaigns benchmarks in the most extreme case if and how much a priori low fidelity knowledge can assist in the discovery campaign. For convenience, we designate SVR-SF$_{boundary}$, RFR-SF$_{boundary}$, and GPR$_{LCB}$-SF$_{boundary}$, SVR-MF$_{boundary}$, RFR-MF$_{boundary}$, and GPR$_{LCB}$-MF$_{boundary}$ (SF denotes single-fidelity, MF denotes multi-fidelity). We gave all the agents a budget of 20 experiment requests in each iteration and simulated each campaign for 100 iterations. In addition, several campaigns have additional stochasticity that requires some thought. More specifically, single-fidelity campaigns with no seed data (i.e. SVR-SF$_{boundary}$, RFR-SF$_{boundary}$, GPR$_{LCB}$-SF$_{boundary}$) create initial seeds data randomly, and random forests also have randomness during the bootstrapping of the samples used in building trees. Even though this stochasticity does not change the candidate acquisition strategy of the agents, it could result in varied campaign performance depending on the inputted random seeds. To account for this, we performed ten trials of campaigns that used those four agents (i.e. all three single-fidelity agents and RFR-SF$_{boundary}$). This helps us look at the overall campaign performance of those agents more objectively because we have better information about the “average” and “variance” in the performance.

Lastly, we bound the performance of our agents above and below by two limiting cases: (1) a perfect agent, where every acquisition is an ideal candidate and the full target space is explored in exactly 202 steps and (2) a naive agent that chooses the next data point from the candidate space at random.

**Figure 5:**
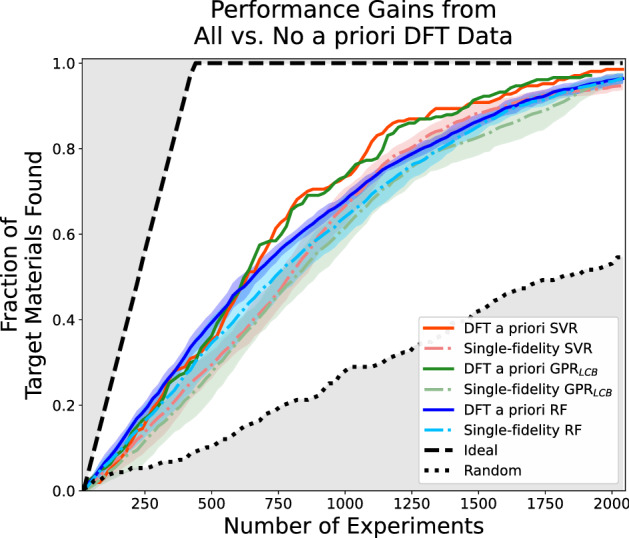
Simulated performance of various campaigns with and without supplying the lower fidelity (DFT) data in the seed. The x-axis corresponds to the high-fidelity (experiments) acquisition budget. The y-axis corresponds to the fraction of ideal materials discovered from the search space. SVR, RF, and GPR$_{LCB}$ correspond to agents using support vector regression, random forests, and Gaussian process regression lower confidence bound, respectively. The shaded colored regions are the standard deviation of materials found for campaigns with multiple trials. The random acquisition and ideal acquisition baselines are also labeled in the figure, representing the lower and upper bounds of agent performance.

Figure 5 shows the results of the simulated discovery campaigns. Where the fraction of the target materials found is plotted against the number of experiments (i.e. high fidelity candidate acquired). The shaded region is the standard deviation of materials found for campaigns with multiple trials. For our initial benchmark, we primarily compare the performance between models. In the single-fidelity case with no access to low-fidelity DFT data, looking at the average target materials found (colored dash lines) in each campaign, random forests agent outperformed support vector regression and Gaussian process regression agents until $\sim$ 850 experiment requests, at which point close to 60% of the ideal candidates had been discovered. Support vector regression agent started outperforming the other two from $\sim$ 850 experiment requests. In the multi-fidelity case where all low-fidelity (DFT) data was made available (colored solid lines), all agents performed similarly (with random forests slightly ahead) until $\sim$550 experiment requests. Afterward, the support vector regression and Gaussian process regression agent outperformed the rest until the end. More importantly, we observed that multi-fidelity agents outperformed their single-fidelity counterparts, demonstrating that these regression algorithms can transfer the knowledge available from the lower-fidelity dataset in making predictions for the high-fidelity target. All of our sequential learning agents consistently outperformed random acquisitions.

**Figure 6:**
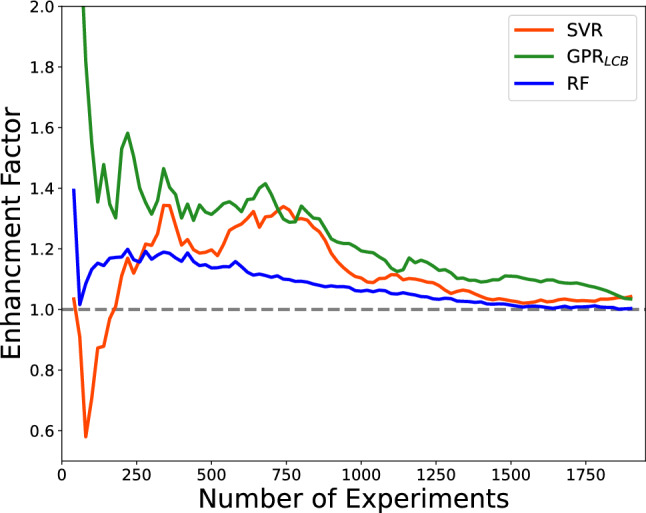
The enhancement factor for quantifying the added value of DFT in the campaigns. The enhancement factors are the ratio of the fraction of discovered materials between the DFT a priori and single-fidelity models at any given budget.

To compare the performance of single and multi-fidelity agents in more detail, we tabulated acceleration factors at 50% and 80% of the total discovery of target candidates in Table 1. To achieve the discovery of 50% of the candidates designed as ideal, multi-fidelity agents reduce the experiments requested by 160, 80, and 180 for support vector regression, random forests, and Gaussian process regression, respectively. At 80% discovery, the acceleration factors are 160, 60, and 220 for support vector regression, random forests, and Gaussian process regression agents, respectively. The enhancement factors shown in Fig. 6 provided a clearer picture of the comparative performance throughout the campaign. We observe that support vector regression multi-fidelity agents briefly underperformed their single-fidelity counterparts in the early stages of campaigns (until $\sim$ 100 experiments). After this point, SVR-MF$_{boundary}$ outperformed SVR-SF$_{boundary}$ by a notable margin to achieve enhancement of a factor of $\sim$ 1.2 to 1.4 until $\sim$ 1000 experiments. This factor diminished slowly as candidates were exhausted for the remainder of the campaign. GPR$_{LCB}$-MF$_{boundary}$ and RFR-MF$_{boundary}$ consistently outperformed their single-fidelity counterpart, with GPR$_{LCB}$-MF$_{boundary}$ having larger enhancement factors. We also notice a similar diminishing trend of their enhancement factors as candidates were exhausted. In summary, all multi-fidelity agents outperformed their single-fidelity counterparts at all points in the process until most target candidates have been acquired. Between the three agents used, support vector regression and Gaussian process regression agents benefited more from a priori data based on the metrics computed.

**Table 1:** Acceleration factor (AF) of multi-fidelity agents in simple acquisitions.

Agents	Single-fidelity experiments performed for 50% discovery	Multi-fidelity AF$_{50\%}$	Single-fidelity experiments performed for 90% discovery	Multi-fidelity AF$_{90\%}$
Support vector regression	800	160	1380	160
Random forest regression	740	80	1360	60
GPR$_{LCB}$	820	180	1360	220

The AFs are the reduction in number of experiments performed by multi-fidelity agents to achieve a certain amount of discoveries. For each row, we highlighted the agents used, the experiments performed by single-fidelity agents to achieve 50% and 80% discovery, and the acceleration factor of the multi-fidelity agents at those discoveries.

### In-tandem acquisitions: both DFT and experiment data are acquired

Having investigated two boundary scenarios in the previous section, with all-or-no low fidelity data, we now turn to our next main question: when and how should we decide to acquire low-fidelity data to support and minimize the number of high-fidelity measurements *during* a sequential, closed-loop data acquisition procedure? To answer this, we simulated another set of campaigns benchmarking single-fidelity versus multi-fidelity. First, to mimic a more true-to-life discovery process, we split the compositions into seed data and candidate data based on their year of discovery according to the ICSD^58^ timeline of their first publication^59^ (Fig.  4). In other words, this rationale for selecting the seed data makes the initial data used for the runs and the successive choice of data by the models entirely deterministic. For single-fidelity campaigns, the data of the first 500 experimentally discovered compositions, up to the discovery year of 1965, were included in the seed data, the remaining (3460 compositions) were included in the candidate data. For multi-fidelity campaigns, the data split was identical, with the addition of corresponding DFT data in each set. Next, we set up the campaigns with a $\epsilon$-greedy agent that used support vectors and a Gaussian processes regression agent (since these two agents had better gains in “Boundary cases: all or no DFT data available”). Therefore, a total of four campaigns were set up: SVR-SF$_{tandem}$, GPR$_{LCB}$-SF$_{tandem}$, SVR-MF$_{tandem}$, and GPR$_{LCB}$-MF$_{tandem}$ (SF denotes single-fidelity, MF denotes multi-fidelity). As before, we also included the two limiting cases of (1) random acquisition and (2) ‘perfect’ acquisition. For the acquisition budget, both SVR-SF$_{tandem}$, GPR$_{LCB}$-SF$_{tandem}$, along with the two limiting cases, had a budget of 5 experiment requests. SVR-MF$_{tandem}$ had a fix-ratio budget of 5 experiments and 5 DFT. GPR$_{LCB}$-MF$_{tandem}$ had a budget of 5 acquisitions, each acquisition can be either experiments or DFT, depending on the uncertainties and *hallucination* of information gained from DFT. Based on optimization results in Supplementary document section S3, $\alpha$=0.08, $\beta$ = 5, and $\gamma$=10 were used for GPR$_{LCB}$-MF$_{tandem}$ to compare against the other sequential learning campaigns. All campaigns were run until 2000 experiments have been acquired, unless it is stopped due to no discovery after 30 iterations (a setting in the campaign hyperparameter).

**Figure 7:**
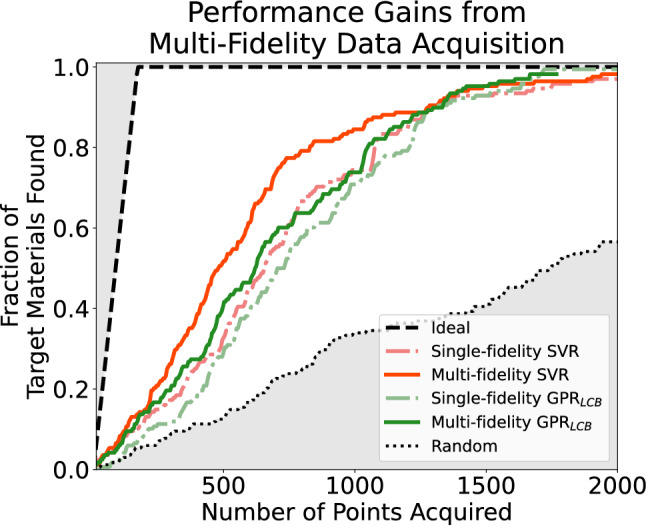
Simulated performance of various campaigns when both DFT and experiment data were acquired during runs. The results of all campaigns are combined in one figure. N$_{experiment}$ on the x-axis corresponds to the experimental acquisition budget. Discoveries on the y-axis correspond to the fraction of ideal materials discovered from the search space. The random acquisition and ideal acquisition baselines are also labeled in the figures.

Figure 7 shows the qualitative results of the simulated campaigns using in-tandem acquisition. Here, SVR-MF$_{tandem}$ agent outperformed its single-fidelity counterpart early in the campaigns (when N$_{experiments}$ reached $\sim$ 100). It then stayed ahead until $\sim$ 1200 experiments were acquired, at which point 90% of the ideal materials had been discovered. GPR$_{LCB}$-MF$_{tandem}$ agent also outperformed its single-fidelity counterpart until 90% of the ideal materials have been discovered (at $\sim$ 1200 experiments). Compared among all four agents, SVR-MF$_{tandem}$ agent’s performance was the best. Furthermore, GPR$_{LCB}$-MF$_{tandem}$ agent’s performance was similar to that of SVR-SF$_{tandem}$’s.

**Figure 8:**
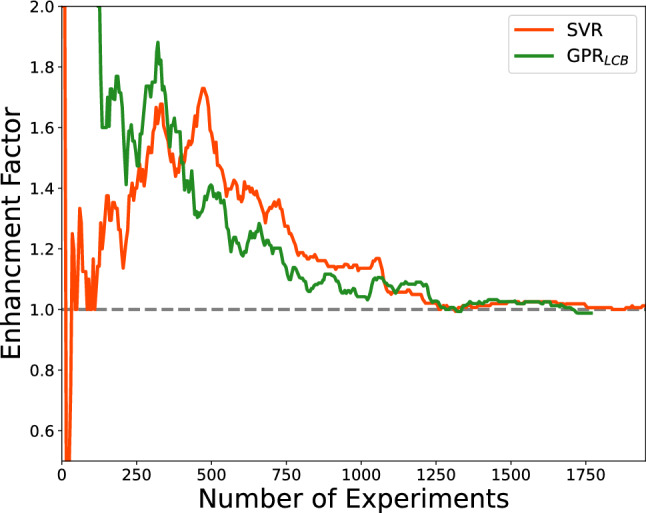
The enhancement factors of in-tandem acquisitions as the campaign progresses.

The acceleration factor (Table 2) of multi-fidelity acquisitions at 50% discovery were 175 and 85 for in-tandem support vector machines and Gaussian processes respectively. At 80% discovery, they were 250 and 159, respectively. The enhancement factors (Fig.  8) of in-tandem multi-fidelity support vector regression is very noisy at first (until N$_{experiments}$ reached $\sim$ 150), which agrees with Fig.  7. Then they stayed above 1 until N$_{experiments}$ reached $\sim$ 1250. The enhancement of GPR$_{LCB}$-MF$_{tandem}$ cannot be calculated at first because its single-fidelity counterpart did not have any discovery. After N$_{experiments}$ reached $\sim$ 100 and its single-fidelity counterpart made some discoveries, the enhancement factors were high but decreased as the acquisition continued and converged to 1 at $\sim$ 1250 experiments.

**Table 2:** Acceleration factor of multi-fidelity agents in in-tandem acquisitions.

Agents	Single-fidelity experiments performed for 50% discovery	Multi-fidelity AF$_{50\%}$	Single-fidelity experiments performed for 80% discovery	Multi-fidelity AF$_{80\%}$
Support vector regression	655	175	1080	250
GPR$_{LCB}$	705	85	1210	159

For each row, we highlighted the agents used, the experiments performed by single-fidelity agents to achieve 50% and 80% discovery, and the acceleration factor (AF) of the multi-fidelity agents. The AF’s are the reduction in number of experiments performed.

## Conclusion

In this work, we develop, implement, and benchmark sequential learning agents that allow for the differentiation of data points of different fidelities. Using our implementation in the CAMD sequential learning framework, we simulated a materials discovery process on previously existing experimental and theoretical electronic band-gap data to inform the selection of these models and suggest hyperparameters that could be used to accompany a ‘real-life’ data acquisition campaign. We found that when all low-fidelity data were provided as a priori knowledge, all multi-fidelity agents outperformed their single-fidelity counterparts and sustained a materials discovery acceleration of 20–60% early on in the campaigns. As the number of experiments acquired in the seed data increased, we saw a decline in additional gain for those multi-fidelity agents. When acquiring low and high-fidelity data in-tandem with support vector regression and Gaussian process regression multi-fidelity agents, both of them still outperformed their single-fidelity counterparts, suggesting strategic acquisitions of lower fidelity data provides a transfer of knowledge and augment higher fidelity target material discovery. We note that, Gaussian process regression multi-fidelity agent here barely outperformed support vector regression single fidelity agent with the settings we provided, which suggests further investigations of the agent.

In summary, we observed a clear trend of multi-fidelity sequential learning agents outperforming those which may only sample at a single-fidelity. The results demonstrate that for studies where low-fidelity data is extremely cheap relative to high-fidelity data, the inclusion of separately labeled data either “up-front” or acquired in-tandem with high-fidelity experiments can increase the rate at which valuable experiments are performed. However, the relative performance of multi-fidelity acquisition is sensitive to the dataset size, ML model selection, and acquisition strategy. Furthermore, the multi-fidelity agents can be extended to have data inputs beyond two fidelities. As mentioned in “Dataset collection and representation”, since the level of fidelity is represented with one-hot encoding, additional fidelity can be passed as an additional column in the feature. Subsequently, acquisition strategies are easily adaptable for multiple levels of fidelity for both of our proposed algorithms by replicating the logic in a nested fashion. For example, for three levels of fidelity, one may acquire the lowest level of fidelity in order to reduce the uncertainty on a median level of fidelity, and acquire a median level of fidelity to reduce the uncertainty of the highest level of fidelity until the experimental budget threshold for new experiments is reached.

Given these dependencies, our framework offers a critical capability that frames automated discovery process itself as an object of study. Our study on multi-fidelity sequential learning campaigns lays a foundation for future research in which both simulations and experiments can be conducted in-tandem with strategies optimized for their relative cost and accuracy.

## Supplementary Information

Supplementary Information.

## Data Availability

The full details of the code are provided in an open-source repository at https://github.com/TRI-AMDD/CAMD.
