# Supplementary material for: Agents for sequential learning using multiple-fidelity data
Source: Sci Rep. 2022 Mar 18;12:4694. doi: 10.1038/s41598-022-08413-8 (PMC8933401; doi:10.1038/s41598-022-08413-8)
Supplement: Supplementary file 1 — Supplementary Information. [file 41598_2022_8413_MOESM1_ESM.pdf]

Supporting Information for "Agents for Sequential Learning Using Multiple-Fidelity Data"

Aini Palizhati[1,2], **Steven B. Torrisi**[1], **Muratahan Aykol**[1], **Santosh Suram**[1], **Jens Hummelshøj**[1], **and Joseph Montoya**[1,*]

[1]Toyota Research Institute, Energy and Materials Division
[2]Carnegie Mellon University, Department of Chemical Engineering
[*]joseph.montoya@tri.global

Section S1: Preliminary model selection

The purpose of the preliminary model selection was to benchmark several ML models for future use and to demonstrate the details of the multi-fidelity framework. Exclusively experimental band gap data was used for model hyperparameter tuning. The initial set of models were support vector regression (SVR), k-nearest neighbors (KNN), and random forest regression (RFR) implemented in scikit-learn[1], and Gaussian process regression (GPR) in GPy[2]. Each model went through separate hyperparameter tuning for us to determine the optimal hyperparameters. For SVR, KNN, and RFR, scikit-learn GridSearchCV was used. For GPR, the kernel function and maximum optimization iteration were adjusted. The best hyperparameters found are in Table S1. The tuned models were then compared against each other using five-fold cross-validation. The evaluation metrics were the average mean absolute error (MAE) and root mean squared error (RMSE) of test data. The prediction accuracy results are in Table S1. We found comparable performance in MAE and RMSE across all four regression models, with the lowest MAE being RF's 0.37 eV and highest being KNN's 0.43 eV; the lowest RMSE was 0.70 eV for RFs and GPs, and the highest was 0.82 for KNN. To put these error values into perspective, we have included some of the literature-reported ML model prediction errors on higher fidelity band gap data. Zhuo *et al.*'s[3] reported test RMSE of 0.45 eV on experimental band gap data. We note that high fidelity data in this paper is aggregated from their work and is a subset of their reported data. Chen *et al.*[4] reported average test MAE of 0.4915 eV on experimental band gap data using their single fidelity model.

Table S1. The tuned hyperparameters and prediction performance of evaluated regressors from scikit-learn[1] and GPy[2]. If a hyperparameter was not explicitly listed, the default was used.

Regressor	Hyperparameter	Test MAE (eV)	Test RMSE (eV)
SVR[1]	kernel=*"rbf"*, degree=3, gamma=*"scale"*, coef0=0.0, tol=0.001, cost constant(C)=10, epsilon=0.1, shrinking=True, cache_size=200, verbose=False, max_iter= -1	0.39	0.72
KNN[1]	n_neighbors=5, weights=*"uniform"*, algorithm=*"auto"*, leaf_size=30, p=2, metric=*"minkowski"*, metric_params=None, n_jobs=None	0.43	0.82
RFR[1]	n_estimators=10, criterion=*"squared_error"*, max_depth=None, min_samples_split=10, min_samples_leaf=1, min_weight$_{f}raction_leaf = 0.0$, max_features=*"auto"*, max_leaf_nodes=None, min_impurity_decrease=0.0, bootstrap=True, oob_score=False, n_jobs=None, random_state=42, verbose=0, warm_start=False, ccp_alpha=0.0, max_samples=None	0.37	0.70
GPR[2]	kernel=RBF kernel, normalizer=None, noise_var=1, optimizer=bfgs, max_iters=200	0.39	0.70

Section S2: Algorithms

Algorithm 1: Epsilon-greedy multi-fidelity agent
Input: Total hypotheses acquisition budget m. High fidelity budget (hf) n, $n \leq m$. Low fidelity (lf) budget $m-n$
Results: m total hypotheses generated, n of which are high fidelity (i.e experimental data).

Given $\hat{y}_i$ for D_i^{hf}, where i = 1, 2, ... N:
    sort D^{hf} by $\hat{y}_i$ reset index;
Initialize hypotheses $H \leftarrow \emptyset$
for i in D^{hf}: **do**
    if high fidelity hypotheses generated < n **then**
        if composition_m has seed data support[a] **then**
            $H \leftarrow H + D_i^{hf}$
    else
        if $n \leq H < m$: **then**
            Find D_j^{lf} that supports[b] D_i^{hf}
            $H \leftarrow H + D_j^{lf}$

---

[a] For each composition, we used l_2 norm to compute its features (generated with matminer[5]) similarity to all other compositions in the seed data. If there was an l_2 norm that is lower than a threshold we set, we considered the candidate composition as similar to some composition(s) in the seed data, thus has seed data support. An l_2 norm of 0 means the lower fidelity measurement already exists in seed data.
[b] For the candidate composition predicted to be ideal but without seed data support, we acquired lower fidelity data with the closest similarity to it. The similarity here was also calculated with the l_2 norm. The number of low-fidelity queries can be specified as a campaign hyperparameter. We chose 1 for our work, meaning whenever the lower fidelity data of the same composition has not been acquired, it gets acquired first.

Algorithm 2: Gaussian process lower confidence bound multi-fidelity agent
Input: Total hypotheses acquisition budget m. For Gaussian process: uncertainty mixing parameter α, uncertainty threshold β, and rank threshold γ.
Results: m total hypotheses generated.

Given $\hat{y}_i$, σ_i for D_i^{hf}, where i = 1, 2, ... N:
    $\hat{z}_i = |\hat{y}_i - y_{ideal}|$;
    $LCB_i = \hat{z}_i - \alpha * \sigma_i$
    Sort D^{hf} by LCB_i, reset index;
Initialize hypotheses $H \leftarrow \emptyset$
for i in D^{hf}: **do**
    while $H \leq m$ **do**
        if $\sigma_i^{hf} < \beta$ **then**
            $H \leftarrow H + D_i^{hf}$
        else
            Hallucinate[a] $\hat{y}_i^{lf}$ to seed data and get LCB_i^*
            Get the new ranking i^* of D_i^{hf}
            if $i^* - i \leq \gamma$ **then**
                $H \leftarrow H + D_i^{hf}$
            else
                $H \leftarrow H + D_i^{lf}$

---

[a] Put the low fidelity prediction into seed data, and ask the agent re-train the ML model. The new model will provide another high fidelity prediction and uncertainty.

Table S2. success rate of agents after 20 iterations of 10 acquisitions with various α. All other acquisition hyperparameters are identical.

**Value of α**	0.01	0.02	0.03	0.04	0.05	0.06	0.07	0.08	0.09
**Discovery**	0.109	0.091	0.123	0.095	0.073	0.118	0.114	0.123	0.095
**Value of α**	0.1	0.2	0.3	0.4	0.5	0.6	0.7	0.8	0.9
**Discovery**	0.059	0.1	0.082	0.091	0.091	0.105	0.114	0.095	0.068
**Value of α**	1	2	3	4	5	6	7	8	9
**Discovery**	0.095	0.064	0.064	0.064	0.045	0.055	0.059	0.059	0.053

Section S3: GPR_{LCB}-MF agent hyperparameter tuning

All optimization results were reported in terms of ALM (one of the performance metrics), which is defined as:

$$ALM(x, N_{exp}) = \frac{\text{Target Materials Discovered after } N_{exp}}{\text{Total Target Materials}} \quad (1)$$

Optimizing α: Given the objective of this work is to evaluate the performance of multi fidelity agents compare to their corresponding single fidelity agents, optimization was performed before the boundary condition acquisition with high fidelity data, and the optimized α was used consistently in all agents (i.e. single and multi-fidelity agents in boundary condition, in-tandem acquisitions). For optimization process, we designed a campaign with various α values. For each campaign, we provided first 500 ICSD[6] reported compositions as seed data, and rest of the compositions as candidate data. Each campaign ran for 20 iterations with a budget of 10 acquisitions per iteration. Based on the results in Table S2, smaller α values resulted in better total number of discoveries from the subset we tested. α=0.08 was selected, as it resulted in the highest number of total discoveries.

Optimizing β and γ: β and γ were used in multi-fidelity in-tandem campaigns, so optimizations were performed before the in-tandem acquisition. We provided the in-tandem multi-fidelity acquisition seed and candidate data to the agent. With $\alpha = 0.08$, we simulated campaigns with combinations of β, γ from $\beta = [5, 10, 20, 30, 40]$ and $\gamma = [0, 5, 10]$. Each campaign ran for 20 iterations with a budget of 10 acquisitions per iteration. Each acquisition can be DFT or experiments. Given the results (Figure S1), we note that a few β with γ=10 performed the best. We selected γ=10 and β=5. Since β is the uncertainty threshold, and we want the agent only acquired experiments when it was more confident.

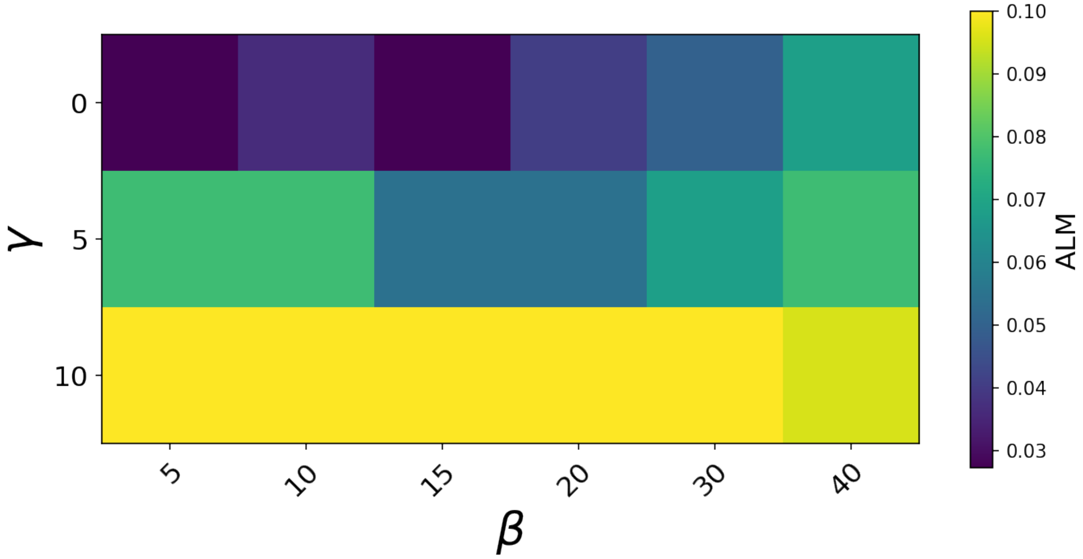

Figure S1. With α=0.08, the heatmap of ALM with respect to various combinations of β and γ.

References

1. Pedregosa, F. *et al.* Scikit-learn: Machine learning in Python. *J. Mach. Learn. Res.* **12**, 2825–2830 (2011).

2. GPy. GPy: A gaussian process framework in python. http://github.com/SheffieldML/GPy (since 2012).

3. Zhuo, Y., Mansouri Tehrani, A. & Brgoch, J. Predicting the Band Gaps of Inorganic Solids by Machine Learning. *J. Phys. Chem. Lett.* **9**, 1668–1673, DOI: 10.1021/acs.jpclett.8b00124 (2018).

4. Chen, C., Zuo, Y., Ye, W., Li, X. & Ong, S. P. Learning properties of ordered and disordered materials from multi-fidelity data. *Nat. Comput. Sci. 2021 1:1* **1**, 46–53, DOI: 10.1038/s43588-020-00002-x (2021).

5. Ward, L. *et al.* Matminer: An open source toolkit for materials data mining. *Comput. Mater. Sci.* **152**, 60–69, DOI: 10.1016/j.commatsci.2018.05.018 (2018).

6. Belsky, A., Hellenbrandt, M., Karen, V., Luksch, P. & IUCr. New developments in the Inorganic Crystal Structure Database (ICSD): accessibility in support of materials research and design. *urn:issn:0108-7681* **58**, 364–369, DOI: 10.1107/S0108768102006948 (2002).
